# Thermal imaging of spin Peltier effect

**DOI:** 10.1038/ncomms13754

**Published:** 2016-12-12

**Authors:** Shunsuke Daimon, Ryo Iguchi, Tomosato Hioki, Eiji Saitoh, Ken-ichi Uchida

**Affiliations:** 1 Institute for Materials Research, Tohoku University, Sendai 980-8577, Japan; 2 WPI Advanced Institute for Materials Research, Tohoku University, Sendai 980-8577, Japan; 3 Center for Spintronics Research Network, Tohoku University, Sendai 980-8577, Japan; 4 Spin Quantum Rectification Project, ERATO, Japan Science and Technology Agency, Sendai 980-8577, Japan; 5 Advanced Science Research Center, Japan Atomic Energy Agency, Tokai 319-1195, Japan; 6 PRESTO, Japan Science and Technology Agency, Saitama 332-0012, Japan

## Abstract

The Peltier effect modulates the temperature of a junction comprising two different conductors in response to charge currents across the junction, which is used in solid-state heat pumps and temperature controllers in electronics. Recently, in spintronics, a spin counterpart of the Peltier effect was observed. The ‘spin Peltier effect' modulates the temperature of a magnetic junction in response to spin currents. Here we report thermal imaging of the spin Peltier effect; using active thermography technique, we visualize the temperature modulation induced by spin currents injected into a magnetic insulator from an adjacent metal. The thermal images reveal characteristic distribution of spin-current-induced heat sources, resulting in the temperature change confined only in the vicinity of the metal/insulator interface. This finding allows us to estimate the actual magnitude of the temperature modulation induced by the spin Peltier effect, which is more than one order of magnitude greater than previously believed.

## Abstract (web summary)

The spin Peltier effect produces a temperature difference along the direction of a spin current. Here, the authors use an active thermography technique to visualize the temperature modulation induced by a spin current injected into a magnetic insulator from an adjacent metal.

In the field of spintronics^1^,^2^,^3^,^4^, the interplay between spin, charge and heat currents has been extensively studied from the viewpoints of fundamental physics and thermoelectric applications^5^,^6^. One of the triggers of this research trend is the discovery of the spin Seebeck effect (SSE)^7^,^8^,^9^,^10^, which refers to the spin current generation in linear response to a temperature gradient. The SSE is usually measured in a junction comprising ferromagnetic and paramagnetic materials; when a temperature gradient is applied across the junction, a spin current is generated due to a non-equilibrium state of spins at the ferromagnet/paramagnet interface^11^,^12^,^13^, which in turn produces a measurable electric field in the paramagnet via the spin–orbit interaction^14^,^15^. The non-equilibrium state is excited by a thermally activated collective dynamics of magnetic moments, that is, magnons^16^, in the ferromagnet, a mechanism different from other spin-transport phenomena driven by conduction electrons^17^,^18^. Owing to the magnon-driven nature, the SSE appears not only in conductors but also in insulators^8^,^10^, expanding the scope of spintronics and thermoelectric technology^19^,^20^,^21^.

The spin Peltier effect (SPE), the reciprocal of the SSE, refers to the heat current generation in linear response to spin current injection. The first observation of the SPE was reported by Flipse *et al*.^22^ using a junction comprising a ferrimagnetic insulator (FI) yttrium iron garnet (YIG) and a paramagnetic metal (PM) Pt; since the SPE appears in insulators, its physics is also discussed in terms of non-equilibrium magnon excitation in the same manner as the SSE^5^,^22^,^23^. Despite its scientific interest and technological importance, except for this demonstration, no experimental studies on the SPE have been reported so far due to difficulty in measuring this phenomenon. The difficulty mainly comes from the length scale of a spin current. Since a spin current disappears within a very short distance typically ranging from several nanometres to micrometres^24^,^25^,^26^, the heat current due to the SPE is generated only in the range of the spin-current decay length. In ref. 22, to detect the temperature change in such a small scale, micro-fabricated thermopile sensors consisting of a number of thermocouples are integrated into a Pt/YIG-based device by using nanolithography. However, to further clarify the behaviour of the SPE, a different approach is necessary.

In the following, we report the thermal imaging of the SPE in PM/FI junction systems, which makes it possible to visualize the spatial distribution of spin-current-induced temperature modulation. This is realized by means of active infrared emission microscopy called lock-in thermography (LIT)^27^,^28^. Although a typical temperature change induced by the SPE is smaller than detection limits of conventional steady-state thermography, the LIT provides a much better signal-to-noise ratio and higher sensitivity with the temperature resolution of <0.1 mK, enabling contact-free measurements of the spatial distribution of the SPE signals over a large area. In the LIT measurements, one inputs a periodic external perturbation, that is, spin current injection in our experiments, to a sample and extracts thermal images oscillating with the same frequency as the perturbation. The obtained thermal images are transformed into the lock-in amplitude and phase images by Fourier analysis. Here the phase image gives the information about the sign of the temperature modulation depending on the periodic perturbation, as well as the time delay due to thermal diffusion. On the basis of this technique, we reveal the distribution of heat sources induced by the SPE.

## Results

### SPE and LIT measurements

**Figure 1:**
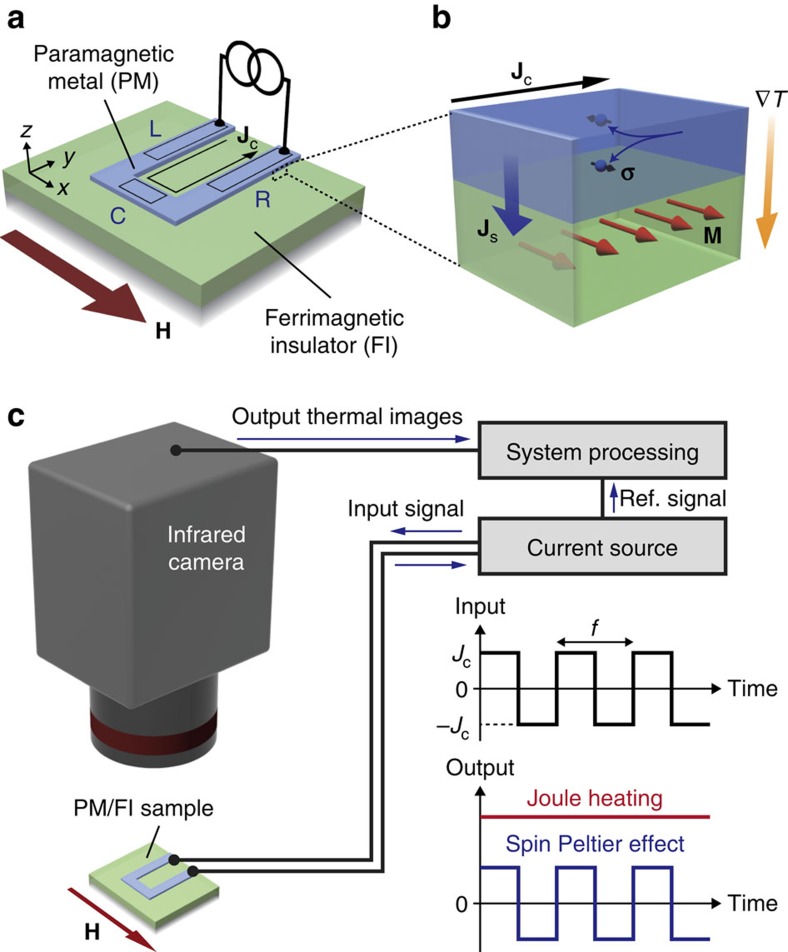
Device structure and experimental set-up. (**a**) A schematic illustration of the sample system used for measuring the SPE. The sample comprises a U-shaped PM (in experiments, Pt or W) film formed on a FI (in experiments, YIG). The squares on PM define the areas L, R and C. (**b**) The SPE induced by the SHE near the PM/FI interface. **H**, **M**, **J**_c_ and **J**_s_ denote the magnetic field vector (with the magnitude *H*), magnetization vector (with the magnitude *M*) of FI, charge current applied to PM and spatial direction of the spin current with the spin-polarization vector **σ** generated by the SHE in PM, respectively. ∇*T*-represents the temperature gradient appearing as a result of the SPE-induced heat current. Owing to the symmetry of the SHE, the **σ** directions on L, R and C are, respectively, along the −*x*, +*x* and −*y* (+*x*, −*x* and +*y*) directions in Pt (W), the spin Hall angle of which is positive (negative). When **M** is along the *x* direction, the SPE appears on L and R because of **M**||**σ**. (**c**) LIT for the SPE measurements. When an a.c. charge current with rectangular wave modulation (with the amplitude *J*_c_ and frequency *f*) is applied to PM, the SPE-induced temperature modulation (∝*J*_c_) oscillates with *f*, while the Joule-heating-induced temperature modulation (∝*J*_c_^2^) is constant in time. The LIT system extracts the first harmonic response of observed thermal images, enabling the pure detection of the SPE free from the Joule-heating contribution.

To excite the SPE, we use the spin Hall effect (SHE)^15^,^24^ as a tool to inject a spin current. Figure 1b shows a schematic illustration of the SHE-induced SPE in a PM/FI junction. When a charge current **J**_c_ is applied to PM with strong spin–orbit interaction, for example Pt or W, the SHE generates a spin current. This spin current then induces spin accumulation near the PM/FI interface, of which the spin-polarization vector **σ** is directed along **J**_c_ × **n** with **n** being the normal vector of the interface plane. When the spin accumulation combines with magnetic moments in FI via interfacial exchange interaction^29^, it transfers spin angular momentum and energy from electrons in PM to magnons in FI, or vice versa, across the interface as spin-transfer torque^1^,^29^. This spin and energy transfer process is proportional to the magnitude of the injected spin current and dependent on whether the **σ** direction in PM is parallel or antiparallel to the magnetization **M** of FI. The non-equilibrium state of the magnon and electron systems induces a heat current **J**_q_ across the PM/FI interface, which satisfies the following symmetry:


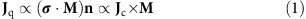


Therefore, by measuring the spin-dependent temperature change in linear response to **J**_c_ near the PM/FI interface, the SPE can be demonstrated.

Figure 1a shows a schematic illustration of the sample system used in this study. The sample consists of a U-shaped Pt or W film formed on an YIG substrate (see Methods for details). To observe the SPE using the LIT technique, we measured the spatial distribution of infrared radiation thermally emitted from the sample surface while applying an a.c. charge current with rectangular wave modulation (with the amplitude *J*_c_ and frequency *f*) to the Pt or W layer and extracted the first harmonic response of detected signals, where we set *f*=5 Hz except for *f*-dependent measurements (Fig. 1c and Supplementary Fig. 1). Here the detected infrared radiation is converted into temperature information through the calibration method detailed in Supplementary Note 1. To enhance infrared emissivity and to ensure uniform emission properties, the sample surface was coated with insulating black ink, of which the emissivity is >0.95. During the LIT measurements, an in-plane magnetic field **H** (with the magnitude *H*) was applied along the *x* direction. When |*H*|>50 Oe, **M** of YIG is aligned along the **H** direction. In our sample, owing to the U-shaped structure of the PM layer, the symmetry of the SHE can be confirmed simultaneously because the relative orientation of **σ** and **M** is different between the areas L, R and C, where **σ**||**M** on L and R and **σ**⊥**M** on C (Fig. 1a,b). Because of the symmetry of the SHE-induced spin-transfer torque^24^,^29^,^30^, the SPE signal can appear on L and R, while it should disappear on C (equation (1)). Since the **J**_c_ and resultant **σ** directions on L are opposite to those on R, the sign of the SPE-induced temperature modulation is reversed between these areas. Importantly, by extracting the first harmonic response of the detected thermal images, we can separate the SPE contribution (∝*J*_c_) from Joule-heating contribution (∝*J*_c_^2^), because the Joule heating generated by the rectangular a.c. current is constant in time as depicted in Fig. 1c. All the measurements were carried out at room temperature and atmospheric pressure.

### Observation of SPE

**Figure 2:**
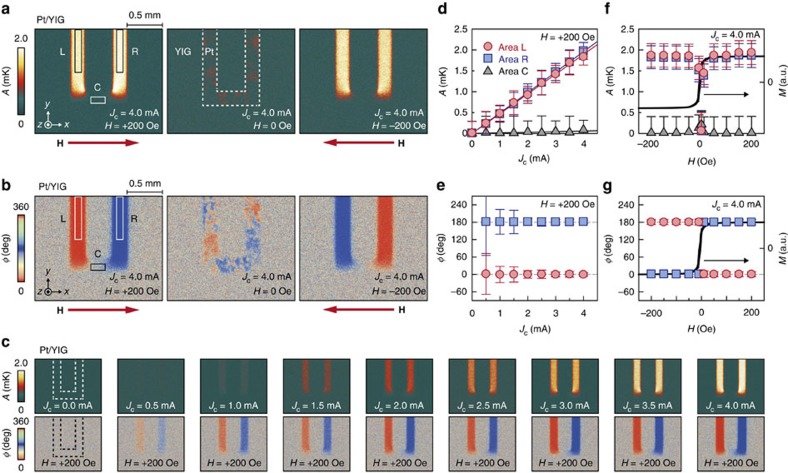
Observation of SPE in Pt/YIG using LIT. (**a**,**b**) Lock-in amplitude *A* (**a**) and phase *ϕ* (**b**) images for the Pt/YIG sample at *J*_c_=4.0 mA. The left, centre and right images were measured at *H*=+200, 0 and −200 Oe, respectively. (**c**) *A* and *ϕ* images for the Pt/YIG sample at *H*=+200 Oe for various values of *J*_c_. (**d**) *J*_c_ dependence of *A* on the areas L (red circles), R (blue squares) and C (grey triangles) of the Pt/YIG sample at *H*=+200 Oe. (**e**) *J*_c_ dependence of *ϕ* on L and R of the Pt/YIG sample at *H*=+200 Oe. (**f**) *H* dependence of *A* on L, R and C of the Pt/YIG sample at *J*_c_=4.0 mA and the *M*–*H* curve (black line) of the YIG. (**g**) *H* dependence of *ϕ* on L and R of the Pt/YIG sample at *J*_c_=4.0 mA and the *M–H* curve of the YIG. The data points in **d**–**g** are obtained by averaging the *A* or *ϕ* values on L, R and C, defined by the squares in the left images of **a**,**b**. The error bars represent the s.d. of the measurements. The lock-in phase does not converge to a specific value when the signal amplitude is smaller than the sensitivity of the LIT; therefore, the *ϕ* data for C are not shown in **e**,**g**.

In Fig. 2a,b, we show the LIT amplitude *A* and phase *ϕ* images for the Pt/YIG sample at *J*_c_=4.0 mA, where the amplitude and phase are defined in the range of *A*>0 and 0°≤*ϕ*<360°, respectively. As shown in the *A* image for *H*=+200 Oe (the left image of Fig. 2a), the clear temperature modulation depending on the **J**_c_ direction appears on L and R, while it disappears on C, which is consistent with the aforementioned symmetry of the SHE (equation (1)). Significantly, the difference in lock-in phases between L and R was observed to be ∼180° (the left image of Fig. 2b); the input charge current and output temperature modulation oscillate with the same (opposite) phase on L (R) in the Pt/YIG sample at *H*=+200 Oe. Since the heat conduction condition is the same between L and R, this *ϕ* shift is irrelevant to the time delay caused by thermal diffusion, indicating that the sign of the temperature modulation on the Pt/YIG surface is reversed by reversing the **J**_c_ direction. We also found that the magnitude of *A* is proportional to *J*_c_, while the *ϕ* shift of ∼180° remains unchanged with respect to *J*_c_ (Fig. 2c–e); the temperature modulation appears in linear response to the charge current applied to the Pt layer.

Next, we measured the *H* dependence of the LIT thermal images using the same Pt/YIG sample. When the **H** direction is reversed and its magnitude is greater than the saturation field, no *A* change and clear *ϕ* reversal appear on L and R (the right images of Fig. 2a,b). At around *H*=0 Oe, the thermal images exhibit patchy patterns corresponding to the magnetic domain structure of YIG, and the net temperature modulation averaged over the Pt/YIG surface disappears (the centre images of Fig. 2a,b). These behaviours result in the odd *H* dependence of the temperature modulation reflecting the magnetization process of YIG (Fig. 2f,g), consistent with the feature of the SPE.

**Figure 3:**
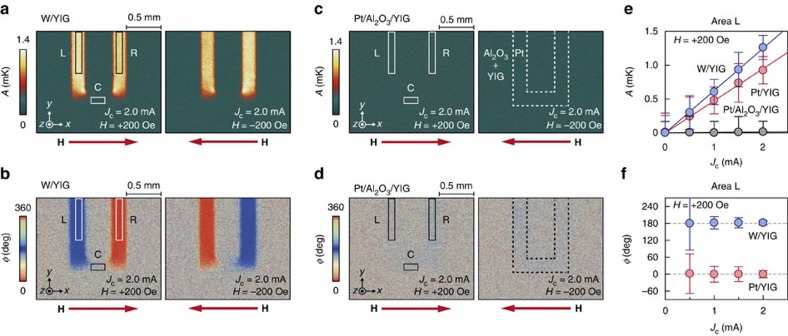
Thermal images in W/YIG and Pt/Al_2_O_3_/YIG. (**a**,**b**) *A* (**a**) and *ϕ* (**b**) images for the W/YIG sample at *J*_c_=2.0 mA, measured at *H*=+200 (left images) or –200 Oe (right images). (**c**,**d**) *A* (**c**) and *ϕ* (**d**) images for the Pt/Al_2_O_3_/YIG sample at *J*_c_=2.0 mA, measured at *H*=+200 or −200 Oe. (**e**) *J*_c_ dependence of *A* on the area L of the Pt/YIG (red circles), W/YIG (blue circles) and Pt/Al_2_O_3_/YIG (gray circles) samples at *H*=+200 Oe. (**f**) *J*_c_ dependence of *ϕ* on L of the Pt/YIG and W/YIG samples at *H*=+200 Oe. The error bars in **e**,**f** represent the s.d. of the measurements.

To further support our interpretation that the current-induced temperature modulation originates from the SPE, we performed control experiments. In Fig. 3a,b, we show the LIT images for the W/YIG sample, where the **σ** direction of the spin current flowing across the W/YIG interface is opposite to that across the Pt/YIG interface since the sign of the spin Hall angle of W is opposite to that of Pt (refs 24, 30). On L and R of the W/YIG sample, the clear temperature modulation was observed. Significantly, the signal in the W/YIG sample is opposite in sign to that in the Pt/YIG sample (compare Figs 2a,b and 3a,b), confirming that the temperature modulation originates from the SHE and spin current injection across the PM/YIG interface. We also checked that the signal disappears in a Pt/Al_2_O_3_/YIG sample, where the Pt and YIG layers are separated by a thin film of insulating Al_2_O_3_ (Fig. 3c,d), indicating an essential role of the direct PM/YIG contact. These experiments, summarized in Fig. 3e,f, clearly show that the temperature modulation near the PM/YIG interfaces is attributed to the SPE driven by the SHE. We note that the sign of the temperature modulation observed here is consistent with the previous experiment^22^ and the sign expected from the SSE^10^.

### Temperature distribution induced by SPE

**Figure 4:**
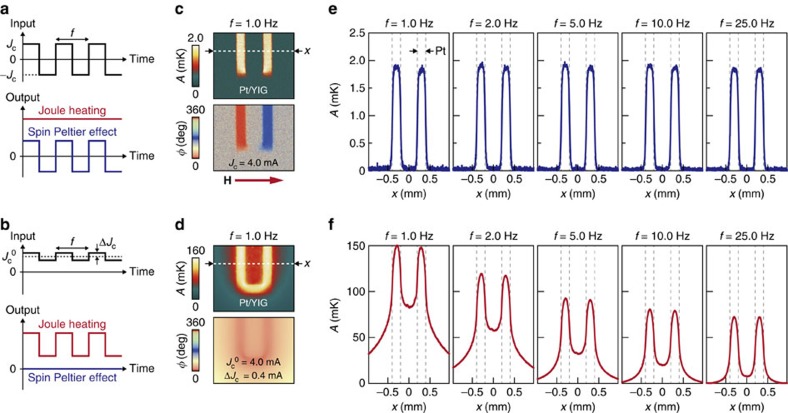
Comparison of temperature distributions induced by SPE and Joule heating. (**a**,**b**) LIT conditions for the SPE (**a**) and Joule-heating (**b**) measurements. In the Joule-heating measurements, a d.c. offset of *J*_c_^0^ and an a.c. charge current with rectangular wave modulation (with the amplitude Δ*J*_c_ and frequency *f*) are applied to PM. In this condition, although both the SPE and Joule-heating signals appear in the first harmonic response of the thermal images, the observed LIT images are governed by the Joule-heating-induced temperature modulation because it is much greater than the SPE signal. (**c**) *A* and *ϕ* images for the Pt/YIG sample in the SPE condition (shown in **a**) at *J*_c_=4.0 mA, *H*=+200 Oe and *f*=1.0 Hz. (**d**) *A* and *ϕ* images for the Pt/YIG sample in the Joule-heating condition (shown in **b**) at *J*_c_^0^=4.0 mA, Δ*J*_c_=0.4 mA, *H*=0 Oe and *f*=1.0 Hz. (**e**) One-dimensional *A* profiles along the *x* direction across the areas L and R of the Pt/YIG sample in the SPE condition at *J*_c_=4.0 mA and *H*=+200 Oe for various values of *f*. (**f**) One-dimensional *A* profiles along the *x* direction across L and R of the Pt/YIG sample in the Joule-heating condition at *J*_c_^0^=4.0 mA, Δ*J*_c_=0.4 mA and *H*=0 Oe for various values of *f*. The SPE-induced temperature distribution is independent of *f*, while the Joule-heating-induced temperature distribution broadens with decreasing *f* due to thermal diffusion.

Now we are in a position to investigate the spatial distribution of the SPE-induced temperature modulation. As already shown in Figs 2 and 3, the SPE signals appear on the PM/YIG interface. Surprisingly, we found that, even under steady-state conditions, the temperature modulation due to the SPE remains confined near the PM/YIG interface without accompanying a temperature change on the areas away from the interface (see Fig. 4c and the temperature profiles measured with low lock-in frequency *f* in Fig. 4e), a situation quite different from thermal diffusion expected from conventional heat sources. To highlight this anomalous behaviour, we compare the temperature distribution induced by the SPE with that induced by Joule heating using the Pt/YIG sample. The temperature modulation induced by Joule heating can be measured by applying a d.c. offset to the rectangular a.c. current, although the Joule-heating contribution is eliminated in the SPE measurements because of the zero offset (Fig. 4a,b). We demonstrated that the Joule heating increases the temperature of the Pt layer irrespective of the **J**_c_ direction and the magnitude of the temperature modulation gradually decreases with the distance from the Pt layer due to thermal diffusion (Fig. 4d,f). This clear contrast between the SPE and Joule-heating signals suggests that the SPE induces non-trivial heat sources near the PM/YIG interfaces.

## Discussion

**Figure 5:**
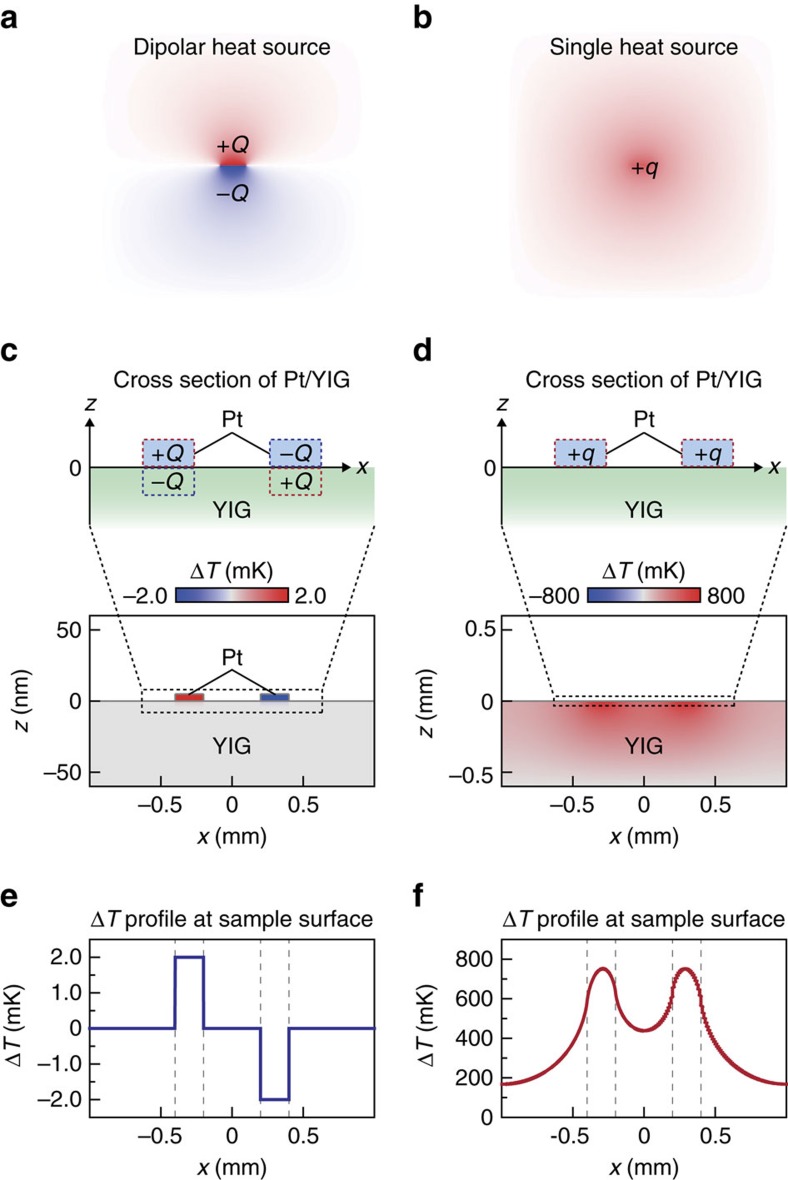
Numerical simulation of temperature distributions. (**a**,**b**) Calculated temperature distributions induced by a dipolar heat source comprising a symmetric pair of a positive component +*Q* and a negative component −*Q* (**a**) and a single heat source +*q* (**b**). The finite element calculations were performed by using the COMSOL Multiphysics software. These distributions were obtained simply by setting the dipolar or single heat source at the centre of a uniform YIG medium. (**c**,**d**) Calculated temperature difference Δ*T* distributions induced by the dipolar heat sources on the Pt/YIG interfaces (**c**) and the single heat sources on the Pt (**d**) of the Pt/YIG model system. The design of the Pt/YIG system is detailed in Supplementary Note 2. The temperature of the bottom of the Pt/YIG system is fixed at 300 K and Δ*T* is defined as the difference from 300 K. Note that the calculation result in **c** is 10^4^ times magnified in the *z* direction to emphasize the temperature distribution confined near the interface. (**e**,**f**) One-dimensional Δ*T* profiles at the sample surface induced by the dipolar heat sources on the Pt/YIG interfaces (**e**) and by the single heat sources on the Pt (**f**) of the Pt/YIG system.

To clarify cross-sectional temperature distribution in the PM/YIG sample, we carry out numerical calculations by means of a two-dimensional finite element method. A model system used for the calculations consists of two Pt films on an YIG substrate, constructed on the basis of the Pt/YIG sample used for the above experiments (see Supplementary Note 2 for details). We calculated steady-state temperature distribution in the *x*–*z* plane in the model system using a standard heat diffusion equation. The temperature distribution induced by Joule heating can be reproduced simply by setting a single heat source on each Pt. As shown in Fig. 5b,d, the single heat source exhibits isotropic temperature change, the magnitude of which gradually decreases with the distance from the Pt. The calculated in-plane temperature profile of the sample surface agrees with the observed Joule-heating-induced temperature distribution (compare Figs 4f and 5f). However, it is obvious that such a standard heat source and its diffusion cannot reproduce the SPE-induced temperature modulation confined near the PM/YIG interfaces.

The spatial distribution of the SPE signals can be explained by assuming the presence of a dipolar heat source, a symmetric pair of positive and negative heat source components, near the PM/YIG interface. Here we calculated the temperature distribution in the Pt/YIG model system with setting dipolar heat sources on the Pt/YIG interfaces, where the polarity of the dipolar heat sources is reversed between the left and right interfaces according to the symmetry of the SHE-induced SPE in our experiments (see the schematic illustration in Fig. 5c). As shown in Fig. 5a, the dipolar heat source exhibits anisotropic temperature change. We found that when such dipolar heat sources are placed near the sample surface, the temperature change is confined in the vicinity of the source positions and the surface-side heat component, placed in Pt, determines the sign of the net temperature change on the interface (Fig. 5c and Supplementary Fig. 2a). This is because the surface-side component accumulates near the PM/YIG interface and generates a greater heating or cooling effect due to small heat dissipation at the sample surface, while the inner-side component, placed in YIG, is diluted due to large heat conduction towards the bulk of YIG. The obtained in-plane temperature profile of the sample surface well reproduces the SPE-induced temperature distribution (compare Figs 4e and 5e and recall that in the LIT measurements, the amplitude is defined in the range of *A*>0 and the phase provides sign information). Importantly, this behaviour appears only when the amounts of the positive and negative components of the dipolar heat sources are exactly the same as each other, where the net heat amount is macroscopically cancelled out; if the net heat amount is finite, the temperature change shows large thermal diffusion like the Joule-heating-induced temperature distribution (Supplementary Fig. 2b).

The clarification of the SPE-induced temperature distribution allows us to estimate the actual magnitude of the SPE signals. Because of the interfacially confined temperature distribution, the magnitude of the SPE signals on the PM/YIG interface is much greater than that on the bare YIG surface even in the vicinity of the interface. In fact, the amplitude of the SPE signal per unit current density *j*_c_ estimated from the data in Fig. 2d is *A*/*j*_c_=4.7 × 10^−13^ Km^2^ A^−1^ on the Pt/YIG interface, which is more than one order of magnitude greater than the value estimated from the data in ref. 22: 8.3 × 10^−15^ Km^2^ A^−1^ on the YIG surface near the interface. This underestimation arises from the fact that only the temperature of the bare YIG surface was detected in the conventional experiments.

The SPE can be used as temperature controllers or modulators in spintronic devices. Owing to the interfacially confined temperature distribution, the SPE enables pinpoint temperature manipulation, which cannot be realized by conventional methods with large thermal diffusion. In addition to the straightforward potential application, the SPE combined with the LIT technique is applicable to magnetic imaging and magnetometry, since SPE signals reflect interfacial magnetization distribution (see the centre images of Fig. 2a,b, where the visualization of two-dimensional magnetic domain structure is demonstrated). The magnetic imaging based on the SPE is realized also owing to its tiny thermal diffusion, enabling the extraction of local magnetization information. Although the temperature modulation generated by the SPE is still small, it can be enhanced by using PM films with larger spin–orbit interaction for efficient charge-to-spin current conversion, improving the exchange interaction at the PM/FI interface for efficient spin angular momentum transfer, and optimizing the thermal design of the PM/FI junction. We anticipate that the unique capability of the SPE will offer a new direction in spintronic applications and the thermal imaging of thermo-spin phenomena will lead to further development of spin current physics.

## Methods

### Sample fabrication

The single-crystalline YIG with a thickness of 112 μm was grown on a single-crystalline Gd_3_Ga_5_O_12_ (GGG) substrate with a thickness of 0.4 mm by a liquid phase epitaxy method. To improve the lattice matching between YIG and GGG, a part of Y in YIG is substituted by Bi; the exact composition of the YIG crystal is Bi_0.1_Y_2.9_Fe_5_O_12_. The YIG surface was mechanically polished with alumina slurry. The U-shaped Pt and W films with a thickness of 5 nm and a width of 0.2 mm were fabricated on the YIG by a radio-frequency magnetron-sputtering method. The Al_2_O_3_ film with a thickness of 1 nm was prepared by an atomic layer deposition method using trimethylaluminum and ozone as a precursor and an oxidant, respectively. For the LIT measurements, the surface of the samples was coated with insulating black ink, which mainly consists of SiZrO_4_, Cr_2_O_3_ and iron oxide-based inorganic pigments.

### Data availability

The data that support the findings of this study are available from the corresponding author on request.

## Additional information

**How to cite this article**: Daimon, S. *et al*. Thermal imaging of spin Peltier effect. *Nat. Commun.* **7**, 13754 doi: 10.1038/ncomms13754 (2016).

**Publisher's note**: Springer Nature remains neutral with regard to jurisdictional claims in published maps and institutional affiliations.

## Supplementary Material

Supplementary Information Supplementary Figures 1-2, Supplementary Table 1, Supplementary Notes 1-2 and Supplementary References.
