# Supplementary material for: Thermal imaging of spin Peltier effect
Source: Nat Commun. 2016 Dec 12;7:13754. doi: 10.1038/ncomms13754 (PMC5159862; doi:10.1038/ncomms13754)
Supplement: Supplementary Information — Supplementary Figures 1-2, Supplementary Table 1, Supplementary Notes 1-2 and Supplementary References. [file ncomms13754-s1.pdf]

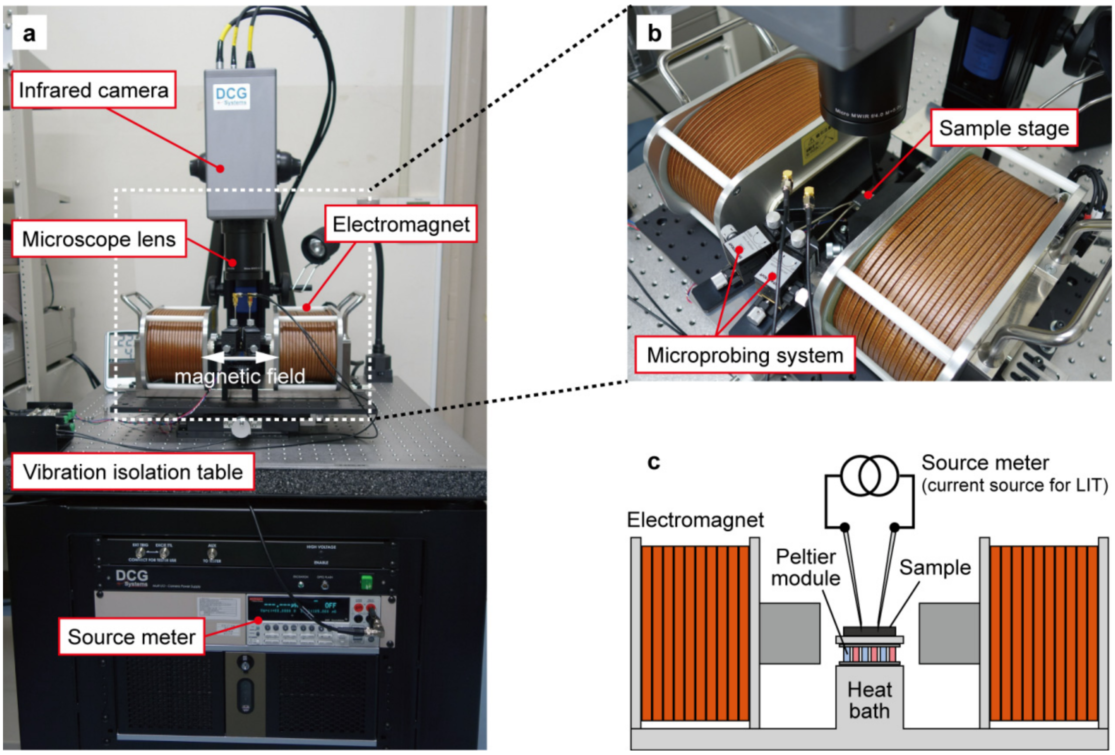


**Supplementary Figure 1 | Lock-in thermography system. a,** An overall picture of the lock-in thermography (LIT) system used in the present study. The LIT system (DCG Systems, ELITE) consists of an infrared camera with an InSb detector and a microscope lens, a system processing unit that performs real-time Fourier analysis of detected thermal images, and a source meter. The spectral range of the InSb detector is 3-5 μm. The spatial resolution of the infrared camera with the microscope lens is ~5 μm. To measure thermal images under an in-plane magnetic field, an electromagnet is installed below the infrared camera. The camera and electromagnet are mounted on a vibration isolation table. **b,** A magnified view of the electromagnet and sample stage. During the LIT measurements, the Pt or W layer of the samples was connected to the output of the source meter via a microprobing system to apply a charge current. **c,** A schematic illustration of the electromagnet and sample stage. The temperature of the sample stage can be varied from 280 K to 320 K by using a thermoelectric Peltier module installed under the stage, where the Peltier module is mounted on a heat bath at room temperature. The temperature difference between the sample stage and the heat bath was measured with a differential thermocouple. This temperature control system was used for the calibration shown in Supplementary Note 1.

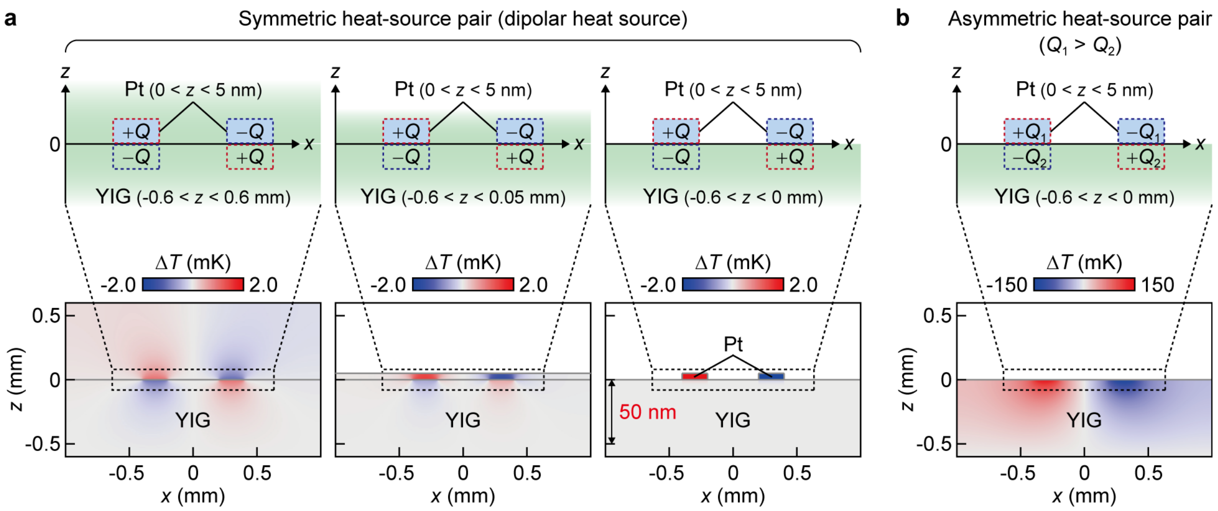


**Supplementary Figure 2 | Temperature distributions induced by symmetric and asymmetric pairs of positive and negative heat sources. a,** Calculated temperature difference $\Delta T$ distributions in the Pt/YIG model systems induced by the dipolar heat sources on the Pt/YIG interfaces for various YIG lengths along the $z$ direction. The dipolar heat source comprises a symmetric pair of a positive component $+Q$ and a negative component $-Q$. $\Delta T$ is defined as the difference from 300 K. The design of the Pt/YIG systems and the boundary conditions are detailed in Supplementary Note 2. We note that, in the Pt/YIG systems used for obtaining the left (center) result in **a**, the Pt is embedded in the YIG, where the YIG is in the range of $-0.6 < z < 0.6$ mm ($-0.6 < z < 0.05$ mm) and the Pt is in the range of $0 < z < 5$ nm. The other calculations were performed by using the Pt/YIG system in which the YIG is in the range of $-0.6 < z < 0$ mm and the Pt is put on the YIG surface. Note that the calculation result for the Pt/YIG system with the YIG of $-0.6 < z < 0$ mm is $10^4$ times magnified in the $z$ direction. **b,** Calculated $\Delta T$ distribution in the Pt/YIG model system induced by asymmetric pairs of positive and negative heat-source components on the Pt/YIG interfaces for the YIG of $-0.6 < z < 0$ mm. The asymmetric heat-source pair comprises the components $+Q_1$ ($-Q_1$) and $-Q_2$ ($+Q_2$) with $Q_1 = 1.01 \times Q_2$. Despite the small difference between $Q_1$ and $Q_2$, the calculated temperature distribution exhibits large thermal diffusion, which is clearly different from the temperature distribution induced by the dipolar heat sources and the experimental results in Fig. 4e. The temperature change is confined near the Pt/YIG interfaces only when $Q_1 = Q_2$.

**Supplementary Table 1 | Material parameters for numerical simulation.**

| | Pt | YIG |
|---|---|---|
| Thermal conductivity ($Wm^{-1}K^{-1}$) | 72 (ref. 1) | 7.4 (ref. 1) |
| Density ($kg\ m^{-3}$) | 21450 (ref. 2) | 5170 (ref. 2) |
| Specific heat ($J\ kg^{-1}K^{-1}$) | 132.56 (ref. 2) | 570 (ref. 2) |

**Supplementary Note 1 | Calibration method for lock-in thermography**

Infrared radiation intensity $I$ thermally emitted from the surface of materials depends on physical properties and surface conditions of the materials. Therefore, in the LIT experiments, the $I$ values detected by the infrared camera need to be converted into temperature $T$ information. This conversion is done by measuring the $T$ dependence of $I$. Since the LIT extracts thermal images oscillating with the same frequency as a periodic external perturbation applied to the sample, the $I$-to-$T$ conversion in the LIT is determined by the differential relation as $\Delta T_{1f}(\mathbf{r}) = dT/dI|_T \, \Delta I_{1f}(\mathbf{r})$, where $\Delta T_{1f}(\mathbf{r})$ and $\Delta I_{1f}(\mathbf{r})$ denote the lock-in responses of the temperature and infrared radiation intensity at the position $\mathbf{r}$, respectively.

In this study, we employed the following five-step calibration method.

(1) Measure the $T$ dependence of $I$ in the steady-state condition by using the infrared camera and the temperature control system shown in Supplementary Fig. 1c,
(2) Calculate the $dT/dI$ function from the obtained $I$-$T$ relation for each pixel,
(3) Perform the LIT measurements; measure the first harmonic response of the $I$ images, i.e. $\Delta I_{1f}$ images, with applying a periodic charge current to the sample,
(4) Determine $T$ values during the LIT measurements for each pixel by using the $I$-$T$ relation and steady-state $I$ images measured in parallel with the $\Delta I_{1f}$ images,
(5) Convert the $\Delta I_{1f}$ images into $\Delta T_{1f}$ images by applying the $dT/dI|_T$ value, obtained from the steps (2) and (4), to each pixel.

This calibration method is valid only when the infrared emissivity of the sample surface is very high. Therefore, the samples used in our experiments were coated with the insulating black ink with the infrared emissivity of > 0.95, which is commercially available from Japan Sensor Corporation. Owing to this high emissivity, the observed thermal images reflect only the temperature distribution of the black ink layer (note that the light transmittance of the black ink layer in the wavelength range of 3-5 μm was observed to be < 0.01).

**Supplementary Note 2 | Procedures and conditions for numerical simulation**

The finite element calculations, shown in Fig. 5 and Supplementary Fig. 2, were performed by means of the COMSOL Multiphysics software. By using the following model systems and boundary conditions, we calculated the steady-state cross-sectional temperature distribution based on a standard heat diffusion equation.

The model system used for the calculations in Fig. 5a,b is a simple YIG medium with a 2 mm × 2 mm square shape. The temperature of the four sides of the YIG square is fixed at 300 K as a boundary condition. In Fig. 5a (5b), we set a dipolar (single) heat source at the center of the YIG square, where the size of the positive and negative components of the dipolar heat source (the size of the single heat source) is 0.2 mm × 5 nm. The thermal conductivity, density, and specific heat of YIG are assumed to be the values shown in Supplementary Table 1.

The Pt/YIG model system used for the calculations in Fig. 5c,d and Supplementary Fig. 2b consists of two Pt rectangles put on an YIG rectangle. The lengths of the YIG rectangle (each Pt rectangle) along the $x$ and $z$ directions are 2 mm (0.2 mm) and 0.6 mm (5 nm), respectively. Here, the bottom of the Pt rectangles is fixed at $z = 0$. The distance between the centers of the Pt rectangles along the $x$ direction is 0.6 mm. To reproduce the experimental situations, we set the following boundary conditions. The top and side surfaces of the Pt/YIG system are connected to air and the temperature of the bottom surface of the YIG is fixed at 300 K. The thermal conductivity, density, and specific heat of each component are assumed to be the values shown in Supplementary Table 1. The interfacial thermal conductance at the Pt/YIG interfaces and the heat-transfer coefficient from the Pt/YIG system to air are set to be $2.79 \times 10^8$ $Wm^{-2}K^{-1}$ (ref. 2) and 10 $Wm^{-2}K^{-1}$ (ref. 3), respectively. In the calculations in Fig. 5c, we set dipolar heat sources on the Pt/YIG interfaces, where one of the components of the dipolar heat sources is placed in the Pt rectangles and the other is in the YIG rectangle. In the calculations in Fig. 5d, we set single heat sources with the same sign in the Pt rectangles. The size of the positive and negative components of the dipolar heat sources and the single heat sources is 0.2 mm × 5 nm. Our numerical calculations confirmed that the macroscopic temperature distributions induced by the dipolar heat sources do not change qualitatively even when the material parameters and the position and size of the heat sources are varied within reasonable ranges. We also checked that the surface temperature profiles of the Pt/YIG system is maintained even when a black-ink coating with a thickness of several tens of micrometers is attached to the Pt/YIG surface.

The numerical calculations in Supplementary Fig. 2a were performed under the same conditions as those used for Fig. 5c except for the YIG length along the $z$ direction. As described in the caption of Supplementary Fig. 2, in the Pt/YIG systems with the YIG of $-0.6 < z < 0.6$ mm and $-0.6 < z < 0.05$ mm, the Pt rectangles are embedded in the YIG. The YIG-length dependence of the temperature distribution clearly shows that, when the dipolar heat sources are placed near the sample surface, the temperature change is confined in the vicinity of the source positions.

**Supplementary References**


1. Uchida, K. *et al*. Longitudinal spin Seebeck effect: from fundamentals to applications. *J. Phys.: Cond. Matter* **26,** 343202 (2014).
2. Schreier, M. *et al*. Magnon, phonon, and electron temperature profiles and the spin Seebeck effect in magnetic insulator/normal metal hybrid structures. *Phys. Rev. B* **88,** 094410 (2013).
3. Bergman, T. L., Incropera, F. P. & Lavine, A. S. *Fundamentals of Heat and Mass Transfer* (John Wiley & Sons., 2011).
